# A randomized controlled trial comparing en-bloc vs lobe-by-lobe HoLEP: surgical efficiency and early continence outcomes

**DOI:** 10.1038/s41391-025-01040-0

**Published:** 2025-11-06

**Authors:** Yahya H. Elmorsy, Ahmed M. Elshal, Ahmed R. EL-Nahas, Ahmed M. EL-Assmy, Mahmoud Laymon

**Affiliations:** Urology department, urology and nephrology center, Mansoura University, Mansoura, Egypt

**Keywords:** Prostatic diseases, Outcomes research

## Abstract

**Objective:**

To compare en-bloc HoLEP with conventional lobe-by-lobe (LBL) HoLEP technique in terms of surgical efficiency, perioperative outcomes, and early continence recovery through a randomized controlled trial.

**Patients and methods:**

This single-center randomized controlled trial included patients with prostate volume >80 mL undergoing HoLEP for bladder outlet obstruction secondary to benign prostatic hyperplasia. Eligible patients were randomized to either en-bloc or LBL HoLEP. All procedures incorporated early apical release and sphincteric mucosal preservation. Assessments were performed preoperatively and at 1, 3, and 6 months postoperatively. primary outcome was enucleation efficiency (resected weight/enucleation time). Secondary outcomes included operative efficiency, laser energy use, blood loss, hospital stay, complications, and functional outcomes (IPSS, QoL, Qmax, PVR, and transient stress urinary incontinence [SUI]).

**Results:**

A total of 123 patients were randomized (en-bloc: 60; LBL: 63). En-bloc HoLEP was associated with shorter enucleation time (62.5 vs. 74.3 min, *P *= 0.02), operative time (78.6 vs. 94.9 min, *P *= 0.0007), and lower laser energy use (135 vs. 154 KJ, *P *= 0.014). Enucleation efficiency was comparable (1.25 ± 0.49 vs. 1.17 ± 0.62 g/min; *P *= 0.42). Both techniques resulted in significant postoperative improvements in IPSS, QoL, Qmax, and PVR (all *P *< 0.0001). Complication rates were similar (14.6% vs. 14%; *P *= 0.8). At 3 months, transient SUI rates were low and comparable (3.8% en-bloc vs. 4% LBL; *P *= 0.3).

**Conclusion:**

En-bloc HoLEP reduces enucleation time, operative time, and laser energy consumption compared to LBL HoLEP, while maintaining comparable safety, efficacy, and early continence outcomes when performed with modern technical refinements.

## Introduction

Since its introduction in 1998 [1], and supported by numerous level 1a evidence studies, Holmium Laser Enucleation of Prostate (HoLEP) has become size-independent gold standard for management of bladder outlet obstruction (BOO) secondary to benign prostatic hyperplasia (BPH) [2–4]. HoLEP is widely accepted as the preferred alternative to open simple prostatectomy for prostates larger than 80 mL [5]. Moreover, when compared to robot-assisted simple prostatectomy, HoLEP has demonstrated several advantages, including shorter operative time, reduced catheter duration and hospital stay, as well as lower postoperative complication rates [6, 7]. There is also accumulating evidence that HoLEP is a feasible and safe surgical option for managing lower urinary tract symptoms (LUTS) in patients with localized and even locally advanced prostate cancer [8]. Furthermore, HoLEP is increasingly utilized in the treatment of recurrent or residual adenoma following prior surgical interventions [9].

Despite being the most extensively studied laser technique for BPH, the adoption of HoLEP remains limited to relatively few centers. This is largely attributed to its steep and prolonged learning curve and absence of structured mentorship programs [10, 11]. Furthermore, the reported prevalence of stress urinary incontinence following HoLEP varies widely from 3.3% to 26% [12, 13].

To improve the learning curve and optimize surgical outcomes, several modifications of the original three-lobe HoLEP technique have been proposed [14, 15]. One notable advancement is the en-bloc enucleation approach incorporating early apical release (EAR), first introduced by Sancha et al. in 2015 using the GreenLight laser. Their technique emphasized early demarcation of the apex with preservation of the mucosal covering of the external urethral sphincter (EUS), aiming to reduce postoperative stress incontinence and improve functional outcomes [16]. These principles of EAR and sphincteric mucosal preservation have since been adapted to the lobe-by-lobe (LBL) technique as well [17].

Although the benefits of EAR and sphincteric mucosal preservation for continence recovery are increasingly recognized, few studies have directly compared en-bloc and lobe-by-lobe HoLEP while consistently applying these refinements. This study aimed to provide high-level evidence on efficacy of en-bloc HoLEP, with specific focus on enucleation efficiency (EE), enucleation time, operative time, and early recovery of continence compared to the conventional lobe-by-lobe approach.

## Patients and methods

### Study design and enrollment

This prospective randomized controlled trial (RCT) was approved by local institutional review board (IRB No.: MS.21.07.1561) and registered on clinicaltrials.gov (Registration ID: NCT07014969)

Inclusion criteria were age ≥40 years and prostate volume between 80 and 200 mL, measured via transrectal ultrasound (TRUS). Surgical indications included refractory lower urinary tract symptoms (LUTS) or complications related to BPH, such as recurrent acute urinary retention, gross hematuria, bladder stones, recurrent infections, or upper urinary tract deterioration. Exclusion criteria included: known neurological conditions affecting bladder function, prostate or bladder cancer, coagulopathy (INR > 1.5 or platelet count <90,000/mL), and ASA score >3. All eligible patients provided informed consent in accordance with Good Clinical Practice and Declaration of Helsinki.

### Randomization

Once HoLEP was indicated, patients were randomized in a 1:1 ratio using computer-generated random tables. To minimize bias, allocation was performed independently by a designated coordinator and was concealed from the outcome assessor.

### Interventions

Both en-bloc and LBL HoLEP procedures were performed using EAR while preserving the mucosa overlying EUS, as previously described [17, 18, 20]. All procedures were performed by four experienced surgeons with substantial experience in HoLEP. The equipment used included a 26 F continuous flow resectoscope (Karl Storz, Tuttlingen, Germany) with rotating inner sheath and Kuntz working element, a 30° optics lens, and a 100 W Sphinx laser system (Lisa Laser, Katlenburg-Lindau, Germany), set at 2 J and 30 Hz (short pulse) for enucleation and 1.5 joules and 15 Hz for coagulation. Tissue morcellation was performed via a 26 F rigid nephoscopy (Karl Storz) using Piranha morcellator system (Richard Wolf, Knittlingen, Germany).

### Preoperative workup

All patients underwent comprehensive clinical evaluation, including medical history, physical examination, International Prostate Symptom Score (IPSS), quality of life (QoL) score, uroflowmetry, post-void residual (PVR) urine measurement, PSA testing, urine analysis, culture, and TRUS. Patients with positive urine cultures received appropriate antibiotics and were included only after documentation of a sterile follow-up culture.

### Outcome measures

The primary outcome was EE, defined as the ratio of the resected tissue weight to the enucleation time (g/min), with enucleation time measured from the insertion of laser fiber to the completion of enucleation. Secondary outcomes included operative efficiency (g/min): resected weight/total operative time, percentage of gland removed: resected weight/total prostate volume, Laser energy density (kJ/g): laser energy used/resected weight, continence recovery, evaluated subjectively via ICIQ-UI SF [21] and objectively using the one-hour pad test. Urinary continence was defined as a negative one-hour pad test and an ICIQ-UI SF score of ≤2. Urinary incontinence was categorized as stress, urge, or mixed, and graded as mild, moderate, or severe based on pad weight [22]. Other variables that were also compared were hemoglobin drop, hospital stay, catheterization time, Clavien-Dindo classified complications. Functional outcomes included IPSS, QoL, Qmax, PVR, and PSA reduction (change from baseline to 6 months post-op, expressed as a percentage).

### Sample size calculation

There was limited published data on enucleation efficiency (EE) specific to en-bloc HoLEP performed with EAR and preservation of the sphincteric mucosa. As a result, enucleation time was adopted as a surrogate measure for enucleation efficiency. Saitta et al. [18] demonstrated a 23% reduction in mean operative time with en-bloc HoLEP with EAR compared to the original three-lobe technique described by Gilling et al. [1], a difference that was assumed to correspond to a comparable increase in EE. Based on our previously published data reporting a mean EE of 1.4 ± 0.6 g/min for the lobe-by-lobe technique [19], we estimated a 23% improvement in the en-bloc group, corresponding to an expected EE of ~1.72 g/min. Assuming a two-sided type I error <5% and 80% statistical power, the minimum required sample size was 56 patients per group. Accounting for a 10% dropout rate, the total target enrollment was set at approximately 120 patients.

### Statistical analysis

Data were analyzed using SPSS version 20 (IBM Corp., Armonk, NY, USA). Categorical variables were compared using chi-square tests. The Kolmogorov–Smirnov test was employed to assess normality of continuous variables. Normally distributed continuous variables were analyzed using Student’s *t* test while non-normally distributed variables were compared using the Mann–Whitney U test for independent samples and the Wilcoxon signed-rank test for paired samples. A *p*-value of less than 0.05 was considered statistically significant.

## Results

**Fig. 1:**
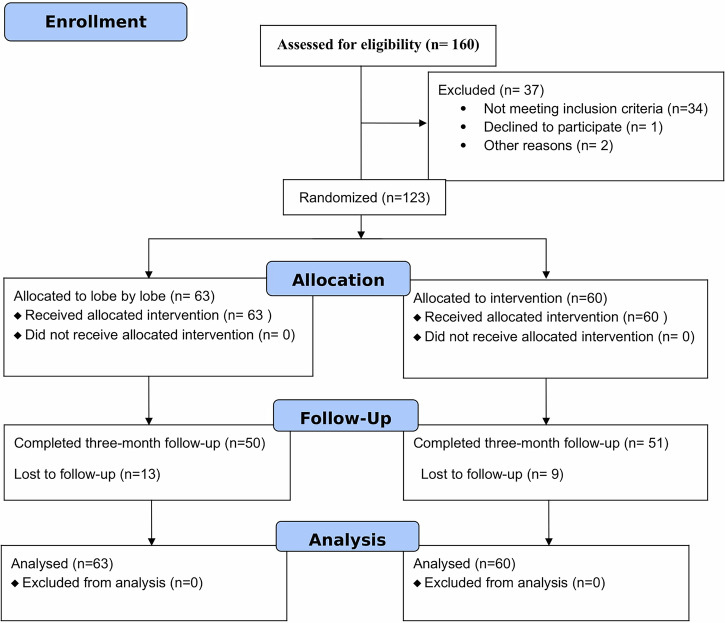
CONSORT flow chart of the study.

Between October 2022 and October 2024, a total of 123 eligible patients were randomly assigned to undergo either en-bloc HoLEP technique (60 patients) or LBL-HoLEP technique (63 patients). The patient flow throughout study is illustrated in Fig. 1. Baseline demographic characteristics were comparable between both groups (Table 1).

**Table 1:** Baseline characteristics of study population.

Variable	Lobe-by-lobe	En-bloc	*P* value
Age: (years): mean ± SD	67.1 ± 7.6	67.2 ± 6.1	0.9^a^
Presentation: *N* (%)			0.59^b^
LUTS	47 (74.7)	49 (81.7)	
Indwelling urethral catheter	12 (19)	9 (15)	
Haematuria	4 (6.3)	2 (3.3)	
BMI (kg/m^2^): mean ± SD	29.3 ± 5.1	29.8 ± 5.2	0.59^a^
Diabetes: *N* (%)	14 (22.2)	17 (28.3)	0.44^b^
Antiplatelet or anticoagulant use: *N* (%)	17 (26.9)	9 (15)	0.27^b^
ASA Score: *N* (%)			0.67^b^
I or II	50 (79.4)	45 (75)	
III	13 (20.6)	15 (25)	
PVR in ml: median (range)	32 (0–575)	20 (0–300)	0.18^c^
PSA in ng/ml: median (range)	7.1 (1.2–28.4)	6.3 (1.1–23.3)	0.25^c^
Preoperative biopsy: *N* (%)	22 (34.9)	21 (35)	0.9^b^
Total prostate volume in ml: mean ± SD	135.2 ± 35.1	127.6 ± 34.2	0.23^a^
Baseline Urine flow parameters for non-catheterized patients:			
IPSS: median (range)	29 (22–35)	26 (21–35)	0.19^c^
QoL: median (range)	6 (4–6)	5 (3–6)	0.34^c^
Q max: ml/sec mean ± SD	8.6 ± 3.8	8.9 ± 3.2	0.64^a^

^a^Independent sample *t*-test.

^b^Chi-square test.

^c^Mann–Whitney test.

### Operative and perioperative parameters

Enbloc was associated with shorter enucleation time (62.5 vs 74.3 min, *p *= 0.02), operative time (78.6 vs 94.9 min, *p *= 0.0007), less total laser energy (135 vs 154 KJ, *p *= 0.014). The mean differences in enucleation and operative times were 11.8 min (95% CI: 2.0–21.6, Cohen’s *d* = 0.43) and 16.3 min (95% CI: 4.6–28.0, Cohen’s *d* = 0.49), respectively, indicating moderate effect sizes. However, enucleation and operative efficiency were comparable between both groups (Table 2). Additionally, there were no statistically significant differences in median hospital stay, time to catheter removal, or perioperative blood loss (Table 2).

**Table 2:** Perioperative efficacy and safety outcomes.

Variables		Lobe-by-lobe	En-bloc	*P* value
Intraoperative measures				
Prostate morphology				0.15^b^
Bilobar (kissing lobes)		28 (44.4%)	35 (58.3%)	
Trilobar (median lobe)		35 (55.6%)	25 (41.7%)	
Concomitant cystolitholapaxy		13 (20.6%)	15 (25%)	0.36^b^
Total LASER Energy (KJ)		154.14 ± 42	135 ± 42.7	**0.014**^**a**^
Operative time (min) mean ± SD median(range)		94.9 ± 33.2 90 (42–180)	78.6 ± 33.1 72.5 (33–160)	**0.007**^**a**^
Enucleation time (min) mean ± SD median(range)		74.3 ± 28 70 (13–150)	62.5 ± 27.3 55 (25–140)	**0.019**^**a**^
Morcellation time (min) mean ± SD median(range)		16.3 ± 12.2 13 (2–60)	13.9 ± 9.5 10 (3–46)	0.216^c^
Enucleated weight (gm) mean ± SD median(range)		76.5 ± 26.5 71 (40–142)	70.5 ± 24.41 68 (27–130)	0.192^a^
Enucleation efficiency (gm/min): mean ± SD		1.17 ± 0.62	1.25 ± 0.49	0.105^a^
Operative efficiency (gm/min): mean ± SD		0.86 ± 0.31	0.98 ± 0.35	0.053^a^
Laser density (KJ/gm): mean ± SD		2.17 ± 0.75	2.02 ± 0.68	0.228^a^
Percentage of enucleated weight/total prostate size		57% ± 15.9%	56% ± 16.7%	0.81^a^
Percent of PSA reduction at 6 months: mean ± SD		81.8% ± 13.7%	86.2% ± 11%	0.46^a^
Intraoperative safety measures				
Capsular violation: *N*. (%)		2 (3.2)	3 (5)	0.68^b^
Bladder injury: *N*. (%)		4 (6.3)	0	0.12^b^
Urethral false passage: *N*. (%)		1 (1.5)	0	1^b^
Need of electrocautery for haemostasis: *N*. (%)		3 (4.7)	1 (1.6)	0.62^b^
Auxiliary TURP for residual tissues: *N*. (%)		1 (1.5)	1 (1.6)	1^b^
Postoperative measures				
Haemoglobin deficit: (gm/dl) median (range)		1.1 (1.2–3.4)	0.7 (1–3.5)	0.098^c^
Catheterization time (days): median (range)		2 (1–7)	2 (1–3)	0.117^c^
Hospital stays (days): median (range)		3 (2–5)	3 (2–5)	0.93^c^
30-day postoperative complications				
Clavien grade				
Failed 1^st^ trial without catheter: *N*. (%)	GI	1 (1.5)	1 (1.8)	1^b^
Need for blood transfusion: *N*. (%)		1 (1.5)	2 (3.3)	0.53^b^
Haematuria: *N*. (%)	GII	1 (1.5)	4 (6)	0.2^b^
Epididymo-orchitis: *N*. (%)	GII	0	1 (1.8)	0.5^b^

Statistically significant *p* < 0.05 values are in bold.

^a^Independent sample *t* test.

^b^Chi-square test.

^c^Mann–Whitney U test.

#### Functional outcomes

**Fig. 2:**
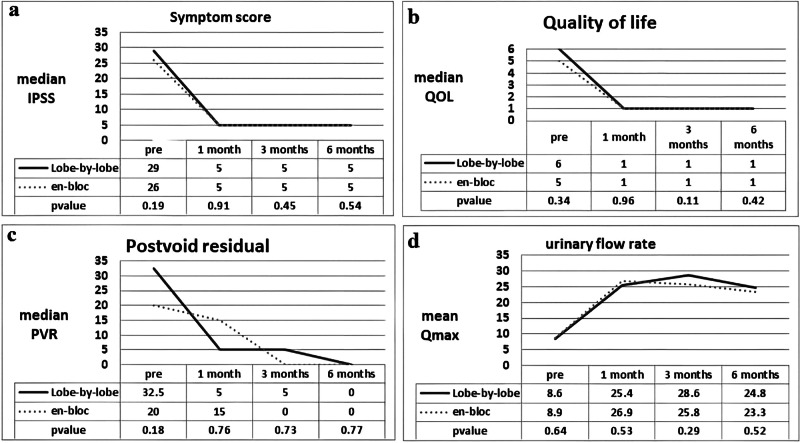
Urinary outcomes among the study groups. **a** IPSS, **b** QoL, **c** PVR and **d** Q-Max.

Both techniques resulted in significant improvements in the International Prostate Symptom Score (IPSS), Quality of Life (QoL), Post-Void Residual (PVR) volume, and maximum urinary flow rate (Qmax) at two weeks, three months, and six months postoperatively when compared to baseline values (*P *< 0.05). No significant differences were observed between two groups at any follow-up visit (Fig. 2). Mean postoperative reduction in prostate-specific antigen (PSA) was comparable between groups.

#### Continence outcomes

The mean ICIQ-UI (International Consultation on Incontinence Questionnaire – Urinary Incontinence) scores among patients who experienced incontinence were comparable between the two groups at both two weeks and three months postoperatively (Table 3).

**Table 3:** Postoperative stress and urge urinary incontinence.

1^st^ follow up visit (2 weeks)			
	LBL	En-bloc	*P* value
Patient-reported stress incontinence: *N*. (%)	3 (5.3)	4 (7.1)	0.11^a^
GI: Mild (≤10 ml)	3 (5.3)	3 (5.3)	
GII: Moderate (11–50 ml)	0	1 (1.8)	
GIII: Severe (>50 ml)	0	0	
ICIQ-UI score median (range)	5 (4–7)	5 (4–14)	1^b^
Patient-reported urge incontinence: *N*. (%)	8 (14.2)	12 (22.2)	0.32^a^
GI: Mild (≤10 ml)	7 (12.5)	8 (14.8)	0.52^a^
GII: Moderate (11–50 ml)	1 (1.7)	3 (5.6)	
GIII: Severe (>50 ml)	0	1 (1.8)	
ICIQ-UI score median (range)	6 (4–10)	7 (5–17)	0.32^b^
2^nd^ follow up visit (3 months)			
	LBL	En-bloc	*P* value
Patient-reported stress incontinence: *N*. (%)	2 (4)	2 (3.8)	0.31^a^
GI: Mild (≤10 ml)	2 (4)	1 (1.9)	
GII: Moderate (11–50 ml)	0	1 (1.9)	
GIII: Severe (>50 ml)	0	0	
ICIQ-UI score median (range)	7 (7–8)	8 (8–9)	1^b^
Patient-reported urge incontinence: *N*. (%)	5 (10)	7 (13.6)	0.96^a^
G0: (negative pad test)	4 (8)	1 (1.9)	0.67^a^
GI: Mild (≤10 ml)	1 (2)	5 (9.8)	
GII: Moderate (11–50 ml)	0	1 (1.9)	
GIII: Severe (>50 ml)	0	0	
ICIQ-UI score median(range)	5 (5–8)	8 (5–10)	0.24^b^

^a^Chi square test.

^b^Mann–Whitney test.

*Transient urge urinary incontinence (UUI)* was observed in 8 patients (14.2%) in the LBL group and 12 patients (22.2%) in the en-bloc group at two weeks. By three months, the incidence had decreased to 5 patients (10%) and 7 patients (13.6%) in the LBL and en-bloc groups, respectively. The differences in UUI incidence and severity between the two groups were not statistically significant. At six months, only two patients in the en-bloc group had persistent UUI, both with mild leakage (pad test weight of 5 g) and ICIQ-UI scores of 11 and 14. These cases were successfully managed with anticholinergic therapy.

*Transient stress urinary incontinence (SUI)* occurred in 3 patients (5%) in the LBL group and 4 patients (7%) in the en-bloc group at two weeks. At three months, this declined to 2 patients (4%) and 2 patients (3.8%), respectively. Again, no significant differences were noted between the two groups in terms of SUI incidence or severity. By six months, persistent SUI was observed in only one patient from each group, with ICIQ-UI scores of 8 (LBL) and 12 (en-bloc). Notably, no patients in either group developed severe SUI (≥Grade III).

#### Complications

The incidence of intraoperative complications, including capsular violation, bladder injury, and urethral false passage, was comparable between the two groups (Table 2). The rates and severity of 30-day postoperative complications were also comparable (Table 2). One patient in each group experienced a failed first trial to void, both of which were managed with re-catheterization for one week, followed by successful voiding. Hematuria requiring catheter prolongation or continuous bladder irrigation (CBI) occurred in 4 patients (6%) in the en-bloc group and in 1 patient (1.5%) in the LBL group.

One patient in the en-bloc group developed a urethral stricture at three months, and another developed a bladder neck contracture at six months. Both complications were managed endoscopically with dilatation and bladder neck incision, respectively.”

#### Outcomes for large prostates (≥150 mL)

Given that prostate size may influence perioperative performance, functional outcomes, and safety, we performed an exploratory subgroup analysis for patients with TRUS-estimated prostate volumes ≥150 mL (*n* = 35; 18 LBL, 17 en-bloc). In this subgroup, the en-bloc group had numerically shorter operative time (92 ± 36.7 vs. 113.5 ± 31 min), shorter enucleation time (71.4 ± 30.4 vs. 84.3 ± 21.4 min), and lower total laser energy use (152.4 ± 41 vs. 166.2 ± 39 kJ) compared with the LBL group. Bladder injury occurred in 4 LBL patients versus none in the en-bloc group, and the need for electrocautery for hemostasis was noted in 3 vs. 1 patient, respectively. At 2 weeks, stress urinary incontinence was observed in 3 en-bloc patients versus none in the LBL group, with only one patient remaining incontinent at 1 month. These data are detailed in (Supplementary Table 1).

## Discussion

The classical three-lobe technique for HoLEP offers the theoretical advantage of a step-by-step enucleation. It divides the procedure into smaller, more manageable steps, while providing clear reference points, borders, and landmarks. This method provides clear anatomical reference points and is particularly useful in prostates with a prominent middle lobe. However, it also has notable limitations. First, it requires three separate incisions, which may increase the risk of leaving residual adenomatous tissue due to inconsistent dissection planes. Additionally, the traditional approach lacks EAR, which increases the risk of mucosal trauma to the EUS. This typically occurs due to downward levering of the scope during the 12 o’clock incision, potentially leading to transient stress urinary incontinence (SUI). The reported incidence of transient SUI after classic HoLEP can reach up to 30% even in the hands of experienced surgeons [13].

To address these issues, en-bloc enucleation with EAR, developed first by Sancha et al. in 2015 using the Green Light Laser [16] was introduced. The potential advantages of this technique include: 1) preserving the integrity of the mucosal covering of the EUS by early demarcation of the ‘white line,’ between the prostatic apex and EUS, 2) dissecting the anterior zone from the sides, lowering the adenoma and eliminating the need for downward levering of the scope, thus avoiding the splitting of the sphincter that can result from the traditional 12 o’clock incision; 3) improved visibility, as irrigation fluid is restricted to a narrower area; and 4) reduced operative time, as the adenoma is dissected apically from the EUS, enabling a circumferential line of dissection that speeds up the procedure because a single plane of dissection is followed throughout the procedure. In 2019, Saitta et al. replicated these principles using HoLEP and reported shorter overall operative time compared to other methods, with a lower incidence of SUI (1.5% at three months) [18].

Some authors have applied the principles of EAR and urethral sphincter mucosal preservation to the lobe-by-lobe technique, reporting improved outcomes, particularly in terms of a reduced incidence of early urinary incontinence [17, 20]. In this study we compared both en-bloc vs lobe by lobe HoLEP while ensuring that both techniques incorporated EAR and careful sphincteric mucosal preservation. This allowed us to isolate the impact of the enucleation strategy itself on perioperative and functional outcomes.

We identified several key findings. First, the en-bloc technique was associated with significantly shorter enucleation and operative times and required less laser energy compared to the LBL approach, despite comparable enucleated tissue weights. These observed intraoperative advantages of en-bloc HoLEP may be attributed to maintaining a consistent dissection plane and avoiding repeated lobe repositioning. While enucleation and operative efficiency were numerically higher in the en-bloc group, these differences did not reach statistical significance. This finding is likely explained by the comparable resected prostate weights between groups, which narrowed the absolute difference in efficiency values. Additionally, variability in operative performance across individual cases—reflected in the relatively wide standard deviations for enucleation time and tissue weight—may have limited the statistical power to detect a significant difference in this derived metric. Importantly, as the primary endpoint (enucleation efficiency) is a derived variable expressed as a ratio (grams/minute), it inherently carries increased variability due to the combination of two independent measures: enucleation time and enucleated weight. This additional variability may have further limited the statistical power to detect a significant difference in this metric, despite clear significant differences in one of its constituent components (enucleation time). Future studies should carefully account for this increased variability when calculating the sample size for derived ratio endpoints.”

Our findings were consistent with previously published data. In a RCT comparing en-bloc vs 2-lobe vs 3 lobe HoLEP, Rucker et al. [23] reported a significantly higher operative efficiency for en-bloc and two-lobe compared to three-lobe (1.82, 1.76 and 1.67 gm/min, respectively *P *= 0.006) with no significant difference between en-bloc and two-lobe techniques. Similarly, Tuccio et al. [24], in a retrospective analysis, found that enucleation time, operative times and laser energy were significantly lower in the en-bloc compared to three-lobe technique. It is however worth noting that the mean prostate volume in the current study was notably larger than in the previous studies.

Second, we evaluated postoperative continence using a robust approach combining objective pad test measurements and subjective ICIQ-SF assessments, allowing a more accurate comparison between en-bloc and lobe-by-lobe (LBL) HoLEP techniques. At three months, the incidence of stress urinary incontinence (SUI) was 3.8% in the en-bloc group and 4% in the LBL group, without significant difference. These rates are notably lower than the historically reported incidence of SUI following conventional HoLEP techniques, where transient SUI rates reached up to 30%. Our findings align with prior studies that implemented similar technical modifications. For instance, Saitta et al. [18] reported a 1.5% SUI rate at three months in 137 patients undergoing en-bloc HoLEP. Similarly, Tuccio et al. [24] compared en-bloc HoLEP with EAR to the lobe-by-lobe technique and found a significantly lower incidence of SUI at one month in favor of the en-bloc (4.5% vs. 13.5%, *P *< 0.05).

In a large multicenter study involving over 5000 patients, the incidence of SUI was significantly higher in the LBL group compared to the en-bloc group. Furthermore, the absence of EAR was identified as an independent predictor of SUI on multivariate analysis [25]. Interestingly, Rucker et al. [23] found no statistically significant difference in SUI incidence among en-bloc, two-lobe, and three-lobe techniques within the first three months (5%, 4%, and 5.5%, respectively; *P *= 0.8), though they used a binary (yes/no) question to assess SUI. The slightly higher SUI rates observed in our study could be attributed to the use of more sensitive assessment methods, including the pad test, which likely captured milder degrees of incontinence.

Although the present study was not specifically powered to detect subtle differences in continence outcomes, our findings suggest that with consistent EAR, continence outcomes are similarly favorable regardless of enucleation technique. Future trials aiming to explore the potential continence benefits of en-bloc HoLEP should be designed as large, multicenter studies, as detecting minor differences may require substantially greater statistical power. Finally, these results support shifting focus from continence—once EAR is standardized—to operative efficiency, precision, and safety as primary endpoints comparing en-bloc and LBL techniques.

Third, we found that both the en-bloc and lobe-by-lobe HoLEP techniques yielded comparable functional outcomes, as reflected by significant and sustained improvements in IPSS, QoL, PVR, and Q max at all follow-up time points. This confirms both techniques effectively relieve obstruction and improve function. Fourth, in the exploratory subgroup analysis of prostates >150 mL, trends favored the en-bloc technique in terms of shorter operative and enucleation times and lower laser energy use. Early stress urinary incontinence at 2 weeks was observed more frequently in the en-bloc group, but this largely resolved by 1 month. Conversely, the lobe-by-lobe group demonstrated a higher frequency of bladder injury and greater use of auxiliary hemostatic procedures. These findings highlight that both techniques have potential advantages and limitations in very large prostates. As the present study was not powered for this subgroup, dedicated trials specifically addressing optimal technique selection in such cases are warranted. Additionally, no significant differences in intraoperative or 30-day postoperative complications were found, suggesting comparable safety profiles when performed by experienced surgeons. These complication rates were also consistent with those reported in previously published studies [26]. Thus, technique selection can depend on surgeon experience without compromising outcomes.

Finally, our study offers several strengths. It is the first randomized controlled trial comparing en-bloc and lobe-by-lobe HoLEP incorporating both Early Apical Release (EAR) and external urethral sphincter mucosal preservation. The use of standardized dual-modality continence assessment—objective (pad test) and subjective (ICIQ-SF)—ensured a comprehensive and reliable evaluation. However, there are some limitations to be acknowledged. First, sexual function was not assessed, as a large proportion of patients were not sexually active preoperatively. Second, the study was conducted at a single high-volume center, and all procedures were performed by experienced endourologists, limiting the generalizability of our findings. Third, although the follow-up period was sufficient to capture early and intermediate outcomes, a longer follow-up is needed. Finally, the study was not powered to detect small differences in early urinary incontinence rates between both techniques. Further multicenter, prospective studies are warranted to assess the learning curve and long-term functional outcomes of different HoLEP techniques.

## Conclusions

This randomized controlled trial demonstrates that both en-bloc and lobe by lobe HoLEP techniques offer comparable functional and safety outcomes. The en-bloc approach, however, confers advantages in enucleation time, operative time and laser energy usage. These findings support the flexibility of surgical technique choice in HoLEP based on surgeon experience, with en-bloc HoLEP representing a streamlined, anatomically guided alternative.

## Supplementary information

Perioperative outcomes of lobe-by-lobe versus En-bloc HoLEP for prostates≥ 150 ml

## Data Availability

The datasets generated during and/or analyzed during the current study are available from the corresponding author on reasonable request.
