# Supplementary material for: A randomized controlled trial comparing en-bloc vs lobe-by-lobe HoLEP: surgical efficiency and early continence outcomes
Source: Prostate Cancer Prostatic Dis. 2025 Nov 6;29(3):551–7. doi: 10.1038/s41391-025-01040-0 (PMC13472966; doi:10.1038/s41391-025-01040-0)
Supplement: Supplementary file 1 [file 41391_2025_1040_MOESM1_ESM.docx]

**Supplementary Table (1): Perioperative outcomes of lobe-by-lobe versus En-bloc HoLEP for prostates**$\boldsymbol{\geq}$ **150 ml**

|  | **Lobe-by-Lobe** | **En-bloc** |
| --- | --- | --- |
| **Age (years): mean ± SD** | 65.4$\pm$11 | 66.3$\pm6.8$ |
| **BMI (kg/m^2^): mean ± SD** | 29$\pm6.3$ | 30.5$\pm5.4$ |
| **PSA (ng/ml): median (range)** | 11.3 $\pm6.7$ | 9.6 $\pm6$ |
| **Total prostate volume in ml: mean**$\boldsymbol{\pm SD}$ | 177.8$\pm14$ | 176.7$\pm16$ |
| **Baseline Urine flow parameters for non-catheterized patients:** | | |
| **IPSS: mean ± SD** | 30.5 ± 5.5 | 27 ±6 |
| **QoL: mean ± SD** | 5.5 ±0.7 | 5.1 ± 0.7 |
| **Q max (ml/sec): mean ± SD** | 10 ±3.3 | 8.5 ± 3 |
| **PVR in ml: median (range)** | 20 (0-80) | 21 (0-200) |
| **Intraoperative Measures** | | |
| **Total LASER Energy (KJ)** | 166.2 ± 39 | 152.4 ± 41 |
| **Operative Time (min) mean ± SD** | 113.5 ±31 | 92±36.7 |
| **Enucleation Time (min) mean ± SD** | 84.3±21.4 | 71.4±30.4 |
| **Enucleated Weight (gm) mean ± SD** | 91.7 ±25 | 89.8±27 |
| **Enucleation Efficiency (gm/min): mean ± SD** | 1.1 ±0.4 | 1.3 ±0.5 |
| **Operative Efficiency (gm/min): mean ± SD** | 0.8 ±0.3 | 1 ±0.3 |
| **Laser density (KJ/gm): mean ± SD** | 0.5 ±0.2 | 0.5 ±0.1 |
| **Intraoperative Safety Measures** | | |
| **Bladder Injury: N. (%)** | 4 (22.2%) | 0 |
| **Need of electrocautery for haemostasis: N. (%)** | 3 (16.7%) | 1 (5.5%) |
| **Auxiliary TURP for residual tissues: N. (%)** | 1 (5.6%) | 0 |
| **Postoperative Measures** | | |
| **Blood transfusion N. (%)** | 1 (5.6%) | 1 (5.9%) |
| **Hemoglobin deficit median (range)** | 1.4 (0.7-2.4) | 1.2 (0.2-1.8) |
| **Length of hospital stay (days): mean ± SD** | 2.7 ±1 | 3 ±0.7 |
| **Postoperative Stress and Urge Urinary Incontinence ( 2 weeks)** | | |
| **Urge incontinence N. (%)** | 3 (16.6%) | 6 (35.2%) |
| **Stress incontinence N. (%)** | 0 | 3 (17.6%) |
| **Postoperative Stress and Urge Urinary Incontinence ( 3 months)** | | |
| **Urge incontinence N. (%)** | 1 (5.5%) | 4 (23.5%) |
| **Stress incontinence N. (%)** | 0 | 1 (6.7%) |
| **Note: Values are presented as mean ± standard deviation or number (percentage). No statistical comparisons were performed for this subgroup, as the analysis was exploratory and the study was not powered for this subset.** | | |
